# Temporal Variations in Respiratory Syncytial Virus Epidemics, by Virus Subtype, 4 Countries

**DOI:** 10.3201/eid2705.204615

**Published:** 2021-05

**Authors:** Lisa Staadegaard, Adam Meijer, Ana Paula Rodrigues, Sue Huang, Cheryl Cohen, Clarisse Demont, Jojanneke van Summeren, Saverio Caini, John Paget

**Affiliations:** Netherlands Institute for Health Services Research (Nivel), Utrecht, the Netherlands (L. Staadegaard, J. van Summeren, S. Caini, J. Paget);; National Institute for Public Health and the Environment (RIVM), Bilthoven, the Netherlands (A. Meijer);; Instituto Nacional de Saúde Doutor Ricardo Jorge, Lisbon, Portugal (A.P. Rodrigues);; Institute of Environmental Science and Research Limited, Upper Hutt, New Zealand (S. Huang);; National Institute for Communicable Diseases, Johannesburg, South Africa (C. Cohen);; University of Witwatersrand, Johannesburg (C. Cohen);; Sanofi Pasteur, Lyon, France (C. Demont)

**Keywords:** Temporal, respiratory syncytial virus, surveillance, epidemiology, epidemics, viruses, the Netherlands, Portugal, New Zealand, South Africa

## Abstract

Temporal variation of respiratory syncytial virus (RSV) epidemics was recently reported to be determined by the dominant RSV subtype. However, when we repeated the analysis for 4 countries in the Northern and Southern Hemispheres, the dominant subtype did not seem to affect temporal variation of RSV epidemics.

Respiratory syncytial virus (RSV) is responsible for most acute lower respiratory tract infections in young children worldwide (*1*) and accounts for a substantial burden among older adults (*2*). Although it is generally accepted that RSV epidemics in temperate climates occur in winter, some temporal variation of epidemics remains unexplained (*3*).

Recently, Yu et al. conducted a study among children (<13 years of age) with pneumonia at the Beijing Children’s Hospital (Beijing, China) during July 2007–June 2015 and reported that temporal variation is partly explained by seasonal differences in virus subtype dominance (*4*). To define the timing of RSV seasonality, they used a regression model and 10% threshold method previously described (*3*). They found that onset and peak of seasons occurred ≈3–5 weeks earlier and that duration was ≈6 weeks longer when RSV subtype A (RSV-A) was dominant than when subtype B (RSV-B) was dominant. These results, if generalizable, would have major implications for the epidemiology of RSV surveillance programs and healthcare planning.

We examined whether similar patterns in the dominant RSV subtype and timing of RSV epidemics were found in the Northern and Southern Hemispheres by using a large dataset from the Global Epidemiology of RSV in the Community and Hospitalised Care study (https://www.nivel.nl/en/geri). We included in our analysis only countries with a temperate climate. For Northern Hemisphere countries, seasons were defined as week 27 through week 26 of the next calendar year; for Southern Hemisphere countries, seasons were defined as week 1 through 52 of the same calendar year. We included seasons if >50 RSV cases with subtyped information available (diagnosed by PCR) had been reported. We included persons of all ages; the Beijing study included only children <13 years of age. In addition, the case definitions for each study did not entirely overlap. In defining the start, duration, and peak of the RSV seasons, we followed a similar approach as Yu et al. (i.e., 10% threshold [*4*]). We defined the onset week of an epidemic as the first of 2 consecutive weeks in which the percentage of specimens testing positive exceeded 10%. The offset week was determined as the second week of the last 2 consecutive weeks when this threshold was breached (*3*).

We explored the relationship between the timing of an epidemic and the dominant RSV subtype (>50% of cases) by calculating the mean start, end, and duration of the seasons according to virus subtype. We applied a regression analysis with robust SEs to account for the potential clustering of individual country results.

We included weekly subtyped RSV data from the Northern (Netherlands and Portugal) and Southern (New Zealand and South Africa) Hemispheres; surveillance systems for those countries are described elsewhere (*5*–*8*). We analyzed 24 seasons (5,189 cases), of which RSV-A was dominant for 14 (Table). A dominant RSV-A or RSV-B season was determined by using the 50% cutoff; this percentage was frequently close to 50%. For example, the proportion of persons with an RSV-A–positive test result was 51%–85% (Figure). All differences in timing were not significant; RSV-A–dominant seasons started 2 weeks earlier (p = 0.3), ended 2 weeks earlier (p = 0.3), and peaked 2 weeks earlier (p = 0.2) than RSV-B–dominant seasons. Mean durations were 14.5 weeks for RSV-A–dominant seasons and 14.9 weeks for RSV-B–dominant seasons (p = 0.9).

**Table:** Summary of seasonal metrics of respiratory syncytial virus epidemics, defined by 10% positivity threshold, by season and country*

Location, season	Start, calendar wk	End, calendar wk	Duration, wk	Peak, calendar wk	No. cases	No. subtyped cases	Subtype A, %	Dominant subtype
Northern Hemisphere								
The Netherlands								
2009–10	49	8	12	3	100	100	44	B
2010–11	4	7	4	6	82	82	68	A
2011–12	51	4	6	51	53	53	36	B
2012–13	51	5	7	2	60	60	75	A
2013–14	3	8	6	4	72	72	44	B
2014–15	6	13	8	9	73	73	37	B
2015–16	51	5	7	2	110	110	35	B
2016–17	47	2	8	51	123	123	70	A
2017–18	47	52	7	51	75	75	17	B
Average	52	6	7	3	83	83	47	B
Portugal								
2012–13	50	1	4	50	94	80	78	A
2013–14	52	3	4	52	298	103	70	A
2014–15	44	18	27	51	412	38	13	B
2015–16	47	13	19	51	646	99	63	A
2016–17	45	12	20	4	682	91	55	A
2017–18	44	15	24	5	1,084	142	51	A
2018–19	44	18	27	10	1,662	101	27	B
Average	47	11	18	2	697	93	51	A
Southern Hemisphere								
New Zealand								
2012	18	37	20	26	880	152	85	A
2013	15	32	18	27	1,238	367	21	B
2014	24	34	11	27	1,406	409	65	A
2015	13	32	20	24	1,430	295	58	A
2016	12	32	21	26	1,020	185	66	A
Average	16	33	18	26	1,195	282	52	A
South Africa								
2016	8	28	21	17	750	675	59	A
2017	7	30	24	16	848	825	33	B
2018	6	26	21	15	922	879	60	A
Average	7	28	22	16	840	793	51	A

**Figure:**
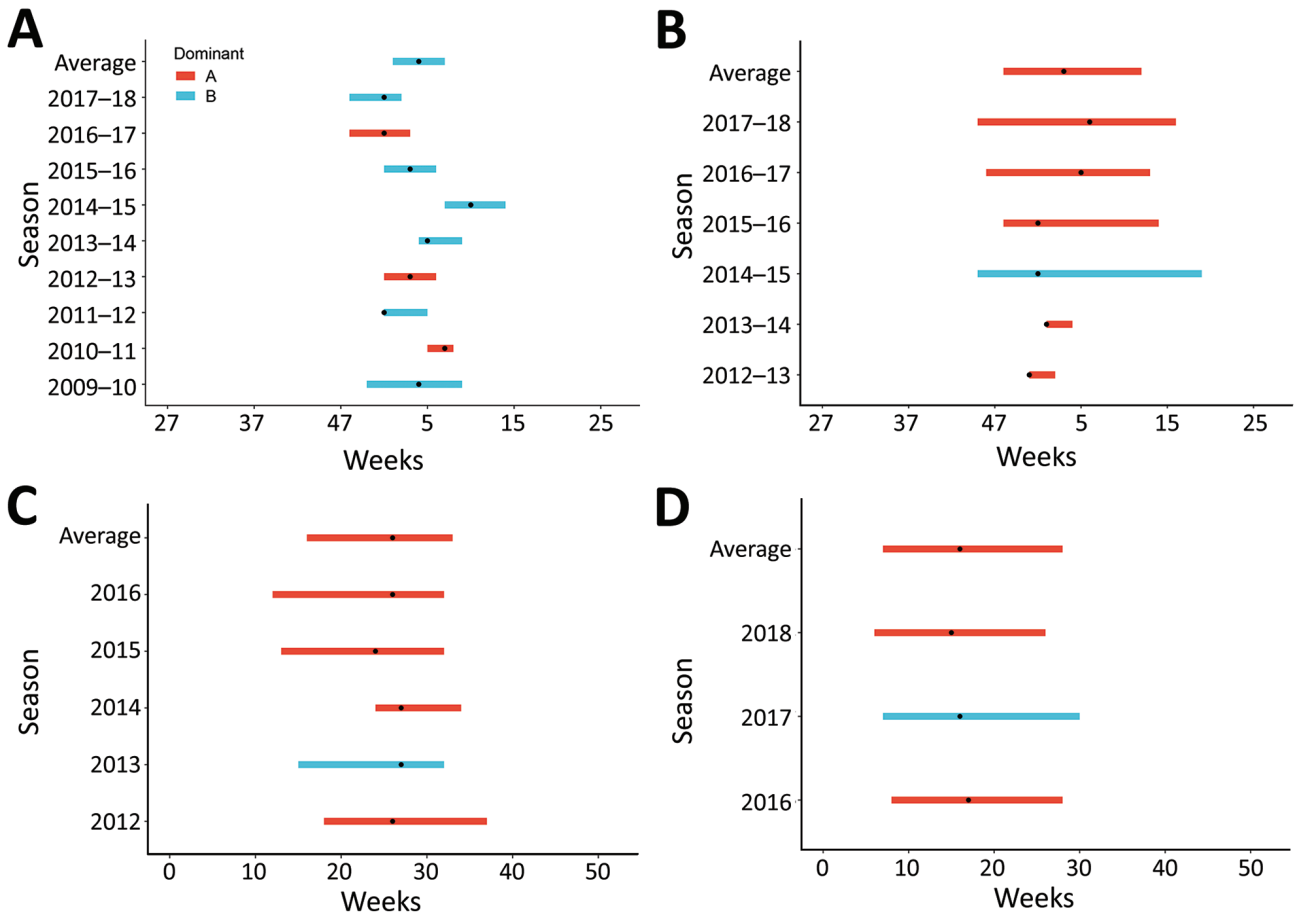
Temporal variation in respiratory syncytial virus, defined by using 10% positivity threshold, by dominant virus subtype, country, and season. A) The Netherlands; B) Portugal; C) New Zealand; D) South Africa. Black dots indicate the peak (highest percentage of cases testing positive) of the season.

We found no significant difference in the effect of the dominant RSV subtype on temporal variation of RSV epidemics. We did not find the earlier start and longer duration of RSV-A–dominant seasons described by Yu et al. when we used similar methods for the countries included in our analysis. Although the national datasets and dataset used by Yu et al. differ from those that we used in several ways (e.g., case definition and age categories), we believe that these differences do not preclude conducting temporal comparisons of this type. 

One limitation of our analysis and that of Yu et al. is the definition of a dominant season. Small differences in virus subtype distribution potentially have a major effect on the results, especially when case numbers are lower in included seasons. An example is the 2016–17 season in Portugal, when RSV-A prevailed but was responsible for only 142 (51%) cases. That finding was similar to that described by Yu et al. for the 2013–14 season, which experienced an almost equal number of cases caused by RSV-A (n = 35) and RSV-B (n = 33). This limitation substantially reduces conclusions that can be drawn from this type of analysis, and we advocate a more stringent definition of an RSV dominant subtype per season (e.g., >70% threshold) for future analyses, thereby ensuring that differences in subtype distribution are real. We recommend that countries monitor RSV subtypes so that our findings can be validated with more data because a temporal variation in RSV epidemics caused by this subtype would have a major effect on the epidemiology of RSV, surveillance programs, and healthcare planning at the local level. 
